# Molecular Conversion of Thermally Stable Cu‐Ag Networks for Mechanically Robust Printed Electronics

**DOI:** 10.1002/advs.78439

**Published:** 2026-10-25

**Authors:** Qikun Feng, Dennis Zhao, Samuel von Keitz, Chris A. Martinez, Abhijit Dasgupta, Hanyu Liu, Zhongxuan Wang, Minhazur Rahman, Nathan Lazarus, Shenqiang Ren

**Affiliations:** ^1^ Department of Materials Science and Engineering University of Maryland College Park Maryland USA; ^2^ Department of Mechanical Engineering University of Maryland College Park Maryland USA; ^3^ Department of Electrical and Computer Engineering University of Delaware Newark Delaware USA

**Keywords:** additive manufacturing, drop tower tests, electrical stability, high‐temperature electronics

## Abstract

Copper–silver inks are promising for printed electronics owing to their high electrical conductivity and oxidation resistance, yet their practical use is limited by silver migration under thermal bias, structural aggregation during curing and sintering, and insufficient mechanical reliability. Here, we report a particle‐free metal–organic reactive ink enabled by an in situ sequential reduction strategy, in which copper is first generated via self‐reduction and subsequently coated with a conformal Ag shell through in situ electroless deposition. This process forms a percolative Ag‐surface‐enriched Cu conductive network without the use of preformed nanoparticles. By eliminating discrete particle precursors, the optimized conductor develops a dense, highly interconnected morphology and achieves a high electrical conductivity of 4.3 MS m^−^^1^. The printed conductors retain stable electrical performance at temperatures up to 800°C and withstand thermal aging in ambient air at 700°C for 48 h. Structural analyses reveal that the conformal Ag shell preserves network continuity and suppresses high‐temperature degradation. This particle‐free strategy avoids the aggregation inherent to conventional particle‐based inks, offering a scalable route to printed conductors with enhanced high‐temperature electrical and mechanical reliability.

## Graphical abstract

An air‐processable Cu–Ag reactive ink enables in situ formation of an Ag‐surface‐enriched Cu conductive network. Optimized Ag distribution suppresses Cu oxidation while preserving continuous electrical pathways, allowing printed conductors to achieve high conductivity, reduced migration, and stable operation at extreme temperatures up to 800 °C in air.

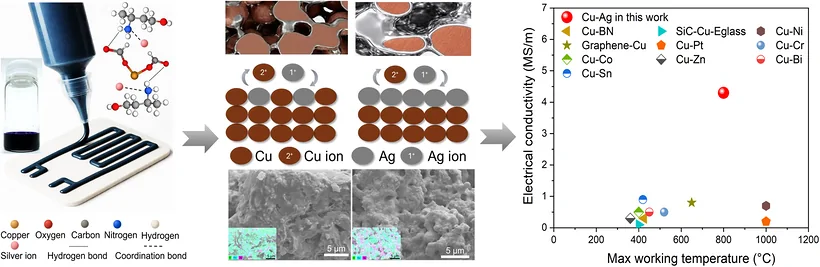

## 1 Introduction

Copper (Cu) has emerged as a cornerstone material for next‐generation electronics owing to its high electrical conductivity and electromigration resistance [1, 2, 3, 4, 5, 6]. Despite its advantages, thermal stability and reliability of copper under oxidative environments remains a fundamental limitation for high‐temperature applications [7, 8, 9]. To address this, substantial efforts have been devoted to suppressing copper oxidation and improving its thermal robustness [10, 11, 12, 13, 14], where the incorporation of alloying elements can significantly enhance oxidation resistance and maintain electrical conductivity at elevated temperatures. However, the formation of copper alloys typically requires high temperatures (>600°C) under inert or reducing atmospheres to achieve phase homogeneity and electrical percolation [15]. Such harsh conditions are incompatible with low‐temperature substrates, limiting the practicality of alloy‐based strategies. An alternative approach is to engineer core–shell copper structures, where a protective shell blocks oxygen diffusion while preserving electrical conduction [16, 17, 18, 19, 20, 21]. Silver (Ag) has emerged as a promising shell material for copper owing to its superior oxidation resistance and electrical conductivity of the core‐shelled Cu‐Ag architectures [22, 23]. For example, Yu et al. synthesized Cu@Ag nanoparticles before ink formulation and investigated their dewetting‐assisted sintering behavior up to 350°C. Although the Ag shell improved the initial oxidation resistance, its thermally activated redistribution progressively reduced shell continuity [24]. Zhang et al. combined preformed Cu particles with an Ag–amino complex and used low‐temperature precuring followed by rapid photonic sintering to produce a Cu‐rich‐core/Ag‐rich‐shell alloy structure, which maintained stable conductivity at 140°C for 500 h [25]. A systematic comparison (Table S1) with representative Cu–Ag hybrid conductors shows that most reported Cu–Ag conductors exhibit excellent conductivity, but their demonstrated high‐temperature electrical stability is typically limited to 300°C in air. Existing Cu–Ag conductors generally rely on preformed metallic particles or complex post‐processing procedures, while thermally induced Ag diffusion, agglomeration, and dewetting can disrupt the protective shell, expose the underlying Cu to oxidation, and ultimately compromise long‐term electrical stability at elevated temperatures. Moreover, the Cu‐Ag particle‐based inks still require high‐temperature or high‐energy processing to achieve sufficient interparticle fusion and electrical percolation [26, 27].

Here, we report a particle‐free strategy to manufacture an Ag‐surface‐enriched Cu conductive network through a single‐step, low‐temperature sintering process in air through a metal‐organic reactive ink platform. In contrast to conventional Cu‐Ag approaches based on pre‐synthesized particles or post‐deposition coating, copper formate and silver nitrate are integrated into one homogeneous reactive ink, in which the diamine ligands are introduced to coordinate Cu species in the presence of silver ions, enabling an in situ cooperative conversion during thermal treatment. Upon heating at 200°C under ambient conditions, copper formate undergoes self‐reduction to generate metallic copper, while Ag^+^ ions are simultaneously reduced and selectively deposited onto the nascent copper surface via electroless galvanic displacement [28, 29, 30], forming a conformal Ag shell around the Cu core. The stoichiometry‐engineered Ag shell effectively suppresses copper oxidation, thereby stabilizing the conductive pathways at elevated temperatures. As a result, the optimized Cu‐Ag conductors achieve a conductivity of 4.3 MS m^−^^1^, maintain stable electrical performance up to 800°C, and withstand 48 h of aging at 700°C in air‐well beyond the conventional thermal limits of copper‐based inks. The advance therefore lies in coupling dual‐precursor sequential conversion with composition‐controlled Ag distribution to access an extreme‐temperature operating regime beyond that of conventional Cu‐Ag printed conductors.

## 2 Results and Discussion

Figure 1a illustrates the conversion of a printable, particle‐free metal‐organic reactive ink into a thermally stable Cu‐Ag conductor. In the reactive ink, copper formate coordinated by diamine ligands serves as the primary copper precursor, while Ag^+^ ions are homogeneously distributed within the ink solution. Upon thermal treatment, the freshly formed copper surface drives an electroless galvanic displacement reaction with neighboring Ag^+^ species, triggering interfacial silver deposition directly onto the Cu surface. This sequential reduction‐displacement process results in the progressive formation of a conformal Ag shell surrounding the copper core. Importantly, the evolution and continuity of the shell are strongly governed by the Cu/Ag molar ratio, which determines the balance between uniform coating and phase segregation. As shown in Figure 1b, the optimized Cu‐Ag conductor integrates multiple performance advantages within a single architecture, achieving a conductivity of 4.3 MS m^−^^1^. Cu‐ceramic composites and Cu‐based alloys can improve thermal durability to some extent, but they typically sacrifice electrical conductivity because the added ceramic phases or alloying elements are less conductive than Cu and may still provide insufficient oxidation protection in high‐temperature air. The conformal Ag shell enhances electrical transport, while simultaneously acting as an effective oxygen‐diffusion barrier that enables stable operation up to 800°C in air. Beyond thermal robustness, the engineered Ag‐surface‐enriched Cu conductive network improves mechanical and electrical reliability, maintaining stable performance under high temperature, high humidity, and high‐g‐force (Figure 1c). These synergistic properties establish a robust foundation for reliable printable electronics capable of stable operation under extreme thermal and electrical stress.

**FIGURE 1:**
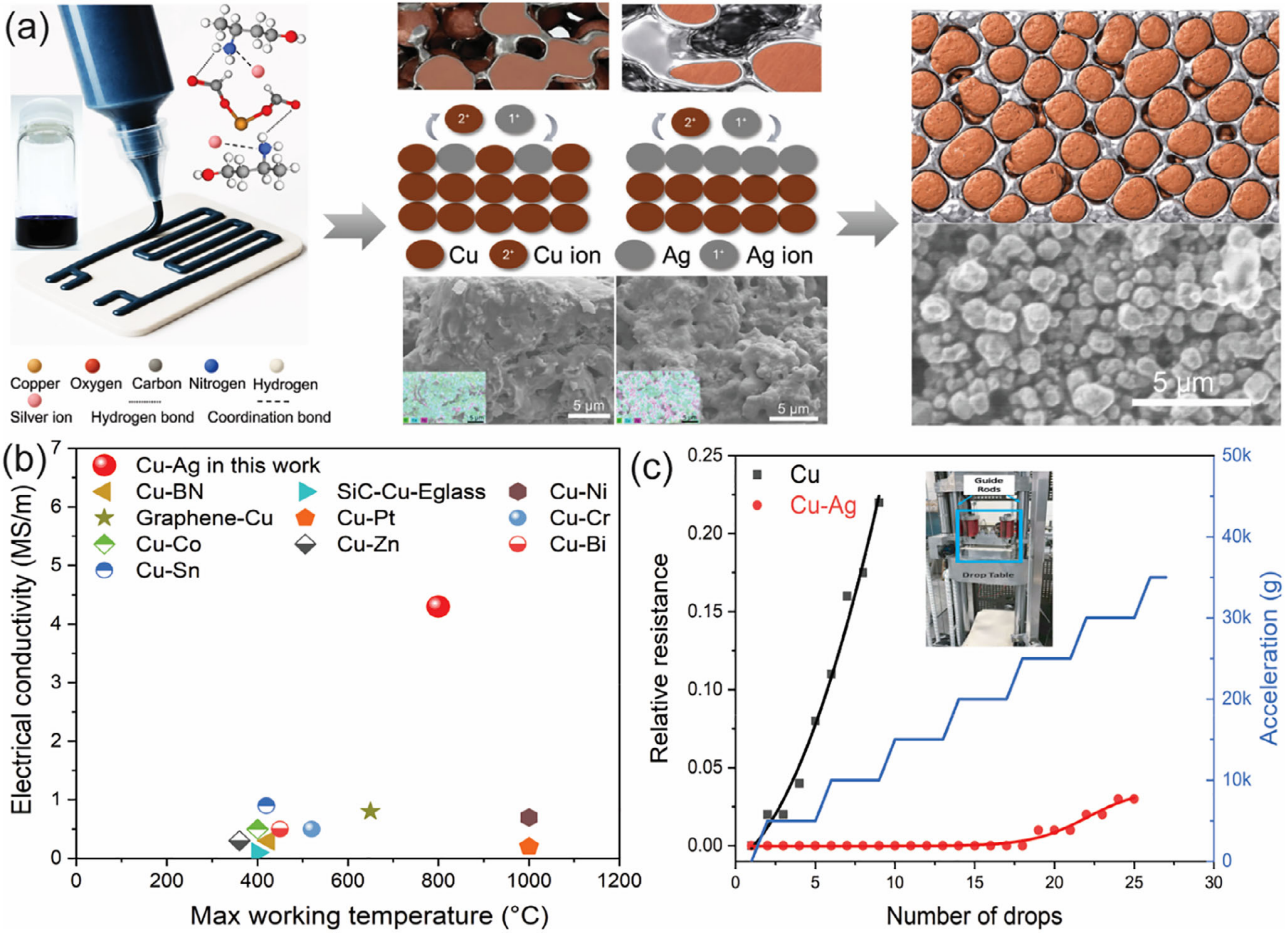
Schematics depicting the experimental procedures followed in this study. (a) Schematic depicting the printable ink and the resulting Ag‐surface‐enriched Cu conductive network. (b) Ashby plot depicting operating temperature vs electrical conductivity reported in the literature and this work [31, 32, 33, 34, 35, 36, 37]. (c) Schematic depicting the high reliability using the Cu‐Ag conductor in a drop tower test.

To elucidate the formation mechanism of the Cu‐Ag structure, the thermal behaviors of individual precursors and mixed inks were first investigated (Figure 2a). Pure copper ink decomposes thermally at approximately 160°C, forming metallic Cu (Figure S1). In contrast, silver nitrate alone decomposes at temperatures above ∼450°C. The AgNO_3_–AMP solution shows a main decomposition temperature at approximately 220–230°C, lower than that of pure AgNO_3_. This result suggests that AMP can coordinate with Ag^+^ and facilitate the low‐temperature decomposition of Ag‐containing species. Notably, when silver nitrate is introduced into the copper‐based ink, the mixed system shows a markedly reduced decomposition temperature of ∼200°C. This pronounced shift suggests that Ag^+^ reduction is not solely governed by the intrinsic thermal decomposition of AgNO_3_, but is instead facilitated by the cooperative effects of the amine ligand and the freshly generated Cu phase. Specifically, AMP can coordinate with Ag^+^ and promote the low‐temperature reduction of Ag‐containing species, while nascent Cu formed from the copper‐based ink can provide active sites for electroless galvanic displacement. Leveraging this thermally coupled reduction pathway, a series of Cu‐Ag inks with different molar ratios were prepared and sintered at 200°C in air. The phase structures of the resulting conductors were analyzed by X‐ray diffraction (Figure 2b). All compositions exhibit distinct diffraction peaks corresponding to Cu (111)/(200) and Ag (111)/(200), confirming the coexistence of crystalline copper and silver after low‐temperature processing. Importantly, room‐temperature conductivity measurements reveal a non‐monotonic, composition‐dependent trend. As the Ag content increases from a low level, the electrical conductivity improves and reaches a maximum value of 4.3 MS/m at an optimized Cu:Ag molar ratio of 1:0.5 (Figure S2). This enhancement cannot be solely attributed to the intrinsically higher conductivity of silver; rather, it arises from the formation of continuous Ag‐surface‐enriched Cu conductive network, in which a conformal Ag shell reduces surface oxidation and interparticle contact resistance while preserving a copper‐dominated percolation network. However, further increasing the Ag fraction leads to a decline in conductivity. Excessive Ag promotes particle‐level segregation and localized aggregation, disrupting interfacial continuity and introducing additional Cu‐Ag boundary scattering that impedes efficient electron transport.

**FIGURE 2:**
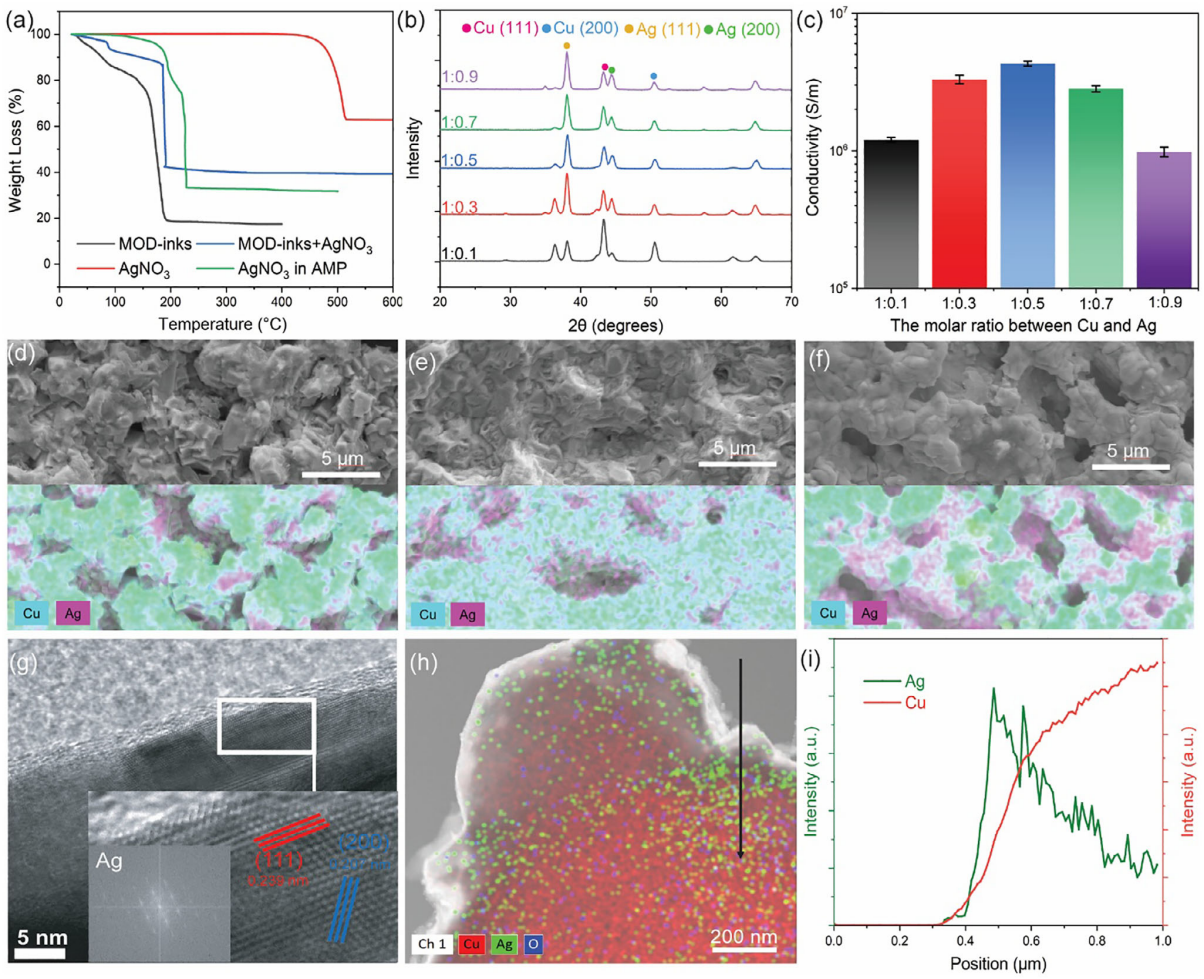
Characterizations of Cu‐Ag conductor with different molar ratios: (a) TGA curves of Cu ink, silver nitrate and mixed inks. (b) XRD patterns of Cu‐Ag conductors sintered at 200°C in air. (c) Conductivity dependence for Cu‐Ag conductor. SEM and corresponding EDX maps of Cu‐Ag conductor with a molar ratio of (d) 1:0.1; (e) 1:0.5; and (f) 1:0.9. (g) TEM, corresponding (h) EDX maps, and (i) the line scan of Cu‐Ag conductor.

Morphological evidence from scanning electron microscopy (SEM) and energy dispersive X‐ray spectrometer (EDX) mapping further supports this interpretation (Figure 2d–f and Figures S3–S5). At a Cu:Ag molar ratio of 1:0.1, only sparse Ag signals are detected on Cu surfaces; at 1:0.5, Ag and Cu distributions largely overlap, indicating uniform shell formation; whereas at 1:0.9, distinct silver‐rich agglomerates are observed. High‐resolution TEM analysis (Figure 2g–i) further confirms the Ag‐surface‐enriched Cu conductive architecture, where lattice fringes corresponding to Ag are clearly resolved at the particle surface, while elemental mapping shows Ag enrichment at the exterior and Cu concentrated in the interior. These results collectively verify the in situ construction of conformal Ag‐surface‐enriched Cu structures via low‐temperature air sintering.

High‐temperature stability of the Cu‐Ag conductors was systematically evaluated through the resistance‐temperature (R‐T) measurements under oxidative conditions (Figure 3a). When the Ag content is relatively low, the conductors undergo oxidation at approximately 500°C, accompanied by a sharp increase in resistance, indicating failure of the protective shell. In contrast, at an optimized Cu:Ag molar ratio of 1:0.5, the conductor exhibits thermal robustness, maintaining stable electrical conduction up to 800°C. This remarkable improvement demonstrates that an optimum Ag shell thickness effectively suppresses oxygen diffusion and protects the copper core at elevated temperatures. However, further increasing the Ag content does not continuously improve oxidation resistance. Although high‐Ag compositions can still retain conductivity before complete failure around 800°C, noticeable resistance fluctuations appear at lower temperatures. This instability is attributed to structural inhomogeneity induced by excessive silver loading. As indicated by SEM and elemental mapping (Figure 2d,e), high Ag fractions promote Cu‐Ag phase segregation and silver aggregation rather than uniform shell formation. Such phase separation leaves portions of copper insufficiently protected, leading to partial oxidation during heating and consequently causing resistance oscillations prior to catastrophic failure. Therefore, the high‐temperature performance of Cu‐Ag conductors is governed not simply by silver content, but by the integrity and continuity of the Ag‐surface‐enriched Cu conductive architecture. The optimized 1:0.5 composition achieves a balanced structure that maximizes oxidation resistance while preserving conductive percolation.

**FIGURE 3:**
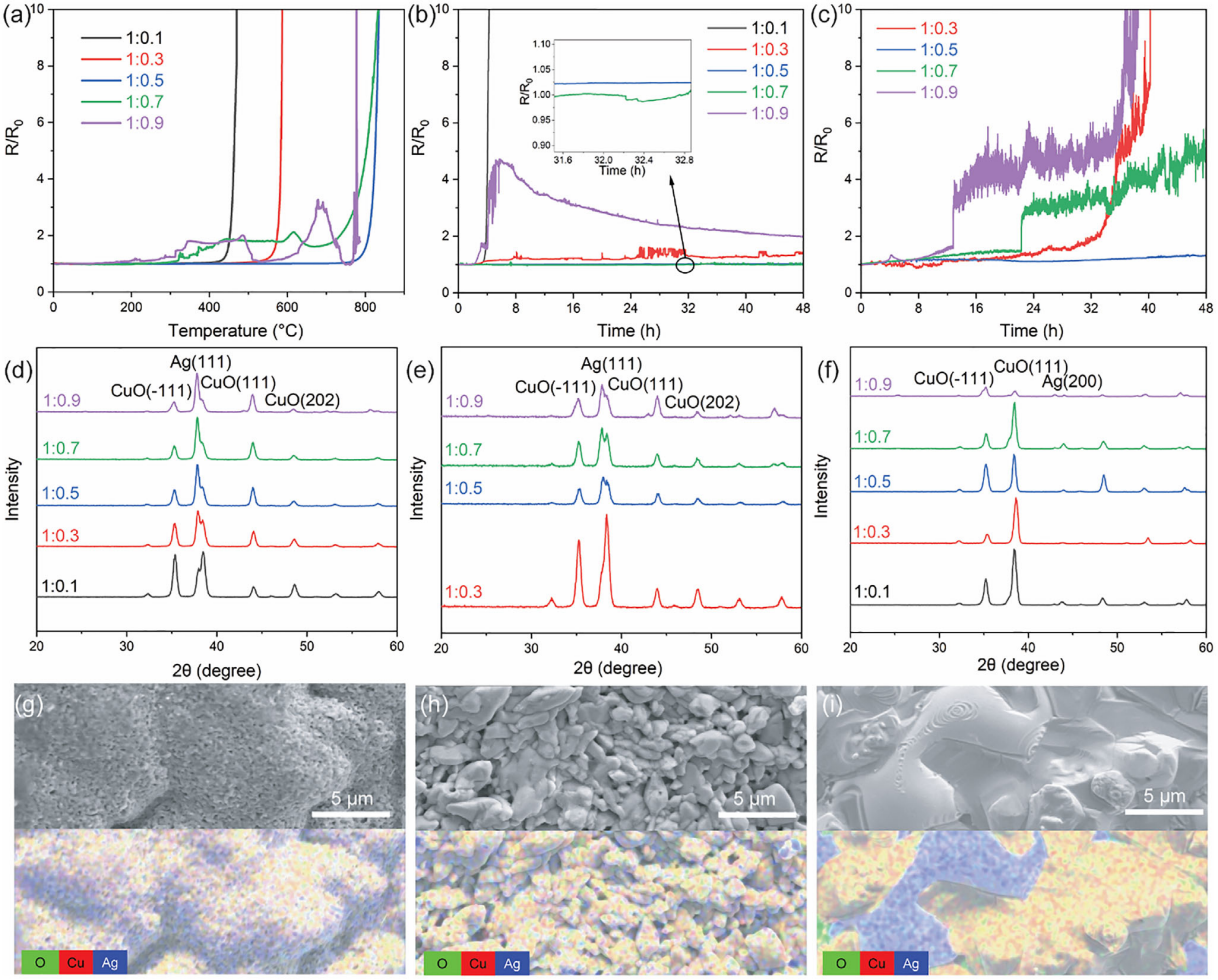
High temperature stability and characterization of Cu‐Ag conductor: (a) The R‐T performance with various molar ratios. (b) Aging test over time at 500°C. (c) Aging test over time at 700°C. XRD patterns of the Cu‐Ag conductor after aging at (d) 500°C; (e) 700°C; and (f) 1000°C. SEM and corresponding EDX maps of the Cu‐Ag conductor (with a molar ratio of 1:0.5) after aging at (g) 500°C; (h) 700°C; and (i) 1000°C.

To further evaluate the thermal durability under prolonged oxidative conditions, isothermal aging tests were conducted at 500°C and 700°C (Figure 3b,c). At 500°C, the Cu‐Ag conductor with the lowest Ag content rapidly loses conductivity due to insufficient surface protection, consistent with its early failure in the R‐T measurement. In contrast, all other compositions retain electrical conduction throughout the aging process. Interestingly, for samples with relatively high Ag loading, the resistance initially increases and subsequently decreases during the 500°C aging test. This non‐monotonic behavior is attributed to residual silver species that remain incompletely reduced after the 200°C sintering step, as evidenced in Figures S6 and S7. Upon exposure to 500°C, these remaining Ag^+^ species undergo further thermal decomposition and reduction, leading to additional silver formation and improved interparticle connectivity, which ultimately lowers the resistance after the initial increase. More stringent evaluation at 700°C further highlights the critical role of compositional optimization. Both insufficient and excessive silver contents result in degraded oxidation resistance under prolonged heating. Low‐Ag conductors fail rapidly due to inadequate oxygen shielding, while high‐Ag conductors exhibit structural instability arising from Cu‐Ag phase segregation and partial exposure of copper regions. Notably, the optimized Cu:Ag molar ratio of 1:0.5 demonstrates exceptional thermal durability, maintaining stable conductivity for at least 48 h at 700°C in air. Precise tuning of the Cu/Ag ratio is therefore essential to achieving long‐term oxidative stability at extreme temperatures.

To elucidate the structural origin of the enhanced thermal stability, the optimized Cu‐Ag conductor (Cu:Ag = 1:0.5) was examined by XRD, SEM and EDX mapping after aging at 500°C, 700°C, and 1000°C (Figure 3d–i and Figures S8–S11). After aging at 500°C, the elemental distributions of Cu and Ag remain highly overlapped, indicating that the Ag‐surface‐enriched Cu conductive architecture is largely preserved. The continuous Ag shell effectively maintains interfacial integrity and protects the copper core from oxidation, consistent with the stable electrical performance observed in the aging test. A similar elemental overlap is observed after aging at 700°C, confirming that the protective shell remains functional even at elevated temperatures. However, compared to the 500°C sample, noticeable morphological evolution occurs. The Cu‐Ag particles exhibit grain growth and partial shape deformation, which can be attributed to thermally activated surface diffusion and coarsening of copper at 700°C. Despite these microstructural changes, the Ag shell provides sufficient coverage to prevent catastrophic oxidation, enabling prolonged electrical stability. In contrast, after aging at 1000°C, the elemental distribution changes dramatically. Clear spatial separation between Cu and Ag is observed, indicating breakdown of the Ag‐surface‐enriched Cu conductive network. This failure is attributed to the fact that the temperature exceeds the melting point of silver (∼962°C). Under such conditions, molten Ag loses interfacial confinement and redistributes away from the copper surface, exposing the underlying Cu to oxidative attack. Consequently, copper undergoes rapid oxidation, leading to electrical failure. X‐ray diffraction analysis (Figure 3d–f) further corroborates this structural evolution. After aging at 500°C and 700°C, distinct diffraction peaks of metallic Ag are present alongside Cu, confirming structural preservation. In contrast, after 1000°C aging, the diffraction pattern is dominated by copper oxide phases, while metallic Ag peaks are largely absent. To quantitatively evaluate the phase evolution, the CuO‐related XRD peak‐ratio analysis was used as a semi‐quantitative oxidation index after high‐temperature exposure. The optimized Cu‐Ag conductor (Cu:Ag = 1:0.5) exhibits a lower CuO/Cu ratio based on the XRD patterns of the Cu‐Ag conductor after aging at 500°C, supporting that appropriate Ag surface/interface enrichment suppresses Cu oxidation and stabilizes the conductive network. These results collectively demonstrate that the thermal stability of the Cu‐Ag conductor originates from the integrity of the Ag shell, which remains effective up to 700–800°C but collapses once the temperature surpasses the melting point of silver.

The mechanical robustness of printed conductors was evaluated using a drop‐tower test under progressively increasing acceleration g‐force (Video S1 and Figure S12). The particle‐based Cu conductor exhibits pronounced resistance fluctuations at approximately 15k g‐force, indicating structural degradation and loss of conductive continuity under drop loading (Figure 4a). In contrast, printed Cu‐Ag conductors from the particle‐free inks shows negligible resistance variation at 15k g‐force and maintains relatively stable electrical performance even under extreme accelerations of 20–30k g‐force (Figure 4b‐c). The superior shock tolerance of the Cu‐Ag conductor originates from its improved interparticle connectivity and structural integrity. For the particle‐based Cu ink processed at 200°C in air, limited sintering results in less dense junctions between particles, which are susceptible to crack initiation and propagation under high mechanical stress. By comparison, the conformal Ag shell formed in the Cu‐Ag architecture enhances interfacial bonding and reduces microstructural defects, leading to a more compact and mechanically robust conductive network. This strengthened percolative framework effectively mitigates crack‐induced resistance fluctuations under high‐g impact conditions. In addition, bending tests were further performed to evaluate the electromechanical stability of the printed conductor under mechanical deformation. The resistance of the conductor remained stable before and after bending, confirming that the Cu‐Ag conductive network maintains electrical continuity under mechanical deformation (Figure S13).

**FIGURE 4:**
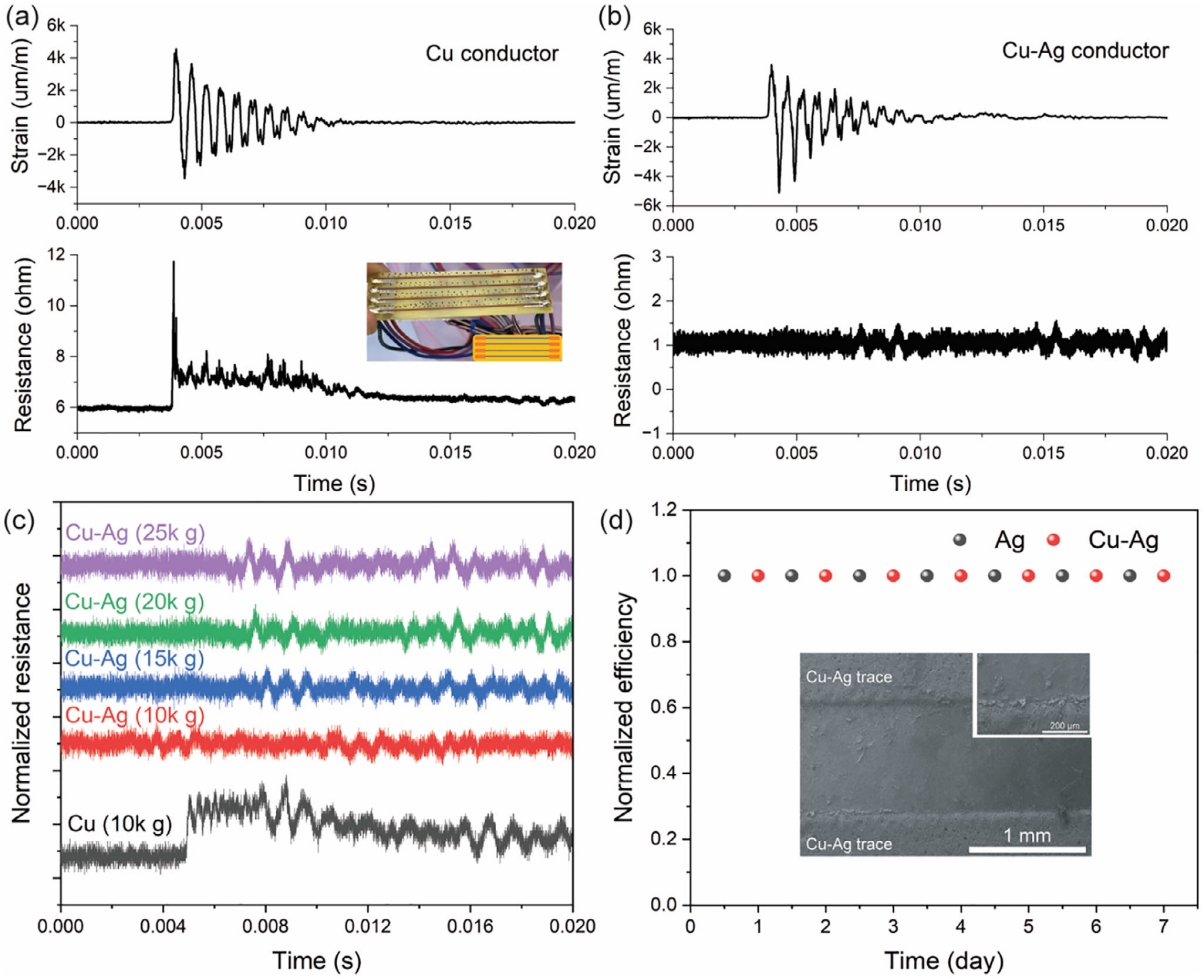
Mechanical shock reliability and electrical reliability tests of Cu‐Ag conductor: (a) Drop tower tests of Cu conductor at 15k g forces. (b) Drop tower tests of Cu‐Ag conductor at 15k g forces. (c) Drop tower tests of Cu and Cu‐Ag conductors at different g forces. (d) Normalized efficiency of solar cells with Ag and Cu‐Ag. (The inserted picture shows the migration of Cu‐Ag trace at 80 V/cm and 100% humidity).

The electrical reliability of the conductors was further evaluated through a migration test performed under identical accelerated conditions of 80 V cm^−^^1^ and 100% relative humidity. Under these harsh humid electrochemical conditions, the pure Ag electrode exhibits severe migration behavior (Figure S14). The SEM analysis reveals that silver species migrate between adjacent electrodes, forming metallic bridges that eventually connect the two electrodes and induce electrical short‐circuiting. This phenomenon is consistent with the well‐known susceptibility of silver to electrochemical migration in high‐humidity, high‐bias environments, where Ag dissolves into ionic species and redeposits under an electric field. In contrast, the Cu‐Ag conductor demonstrates significantly improved resistance to electrical migration. No obvious dendritic growth or inter‐electrode bridging is observed under the same testing conditions. The suppressed migration behavior can be attributed to the engineered Cu‐Ag architecture and modified interfacial chemistry, which reduce the availability of mobile silver species and stabilize the conductive network under electrical bias. As a result, the Cu‐Ag conductor maintains electrical isolation between electrodes, ensuring operational reliability in high‐humidity and high‐field environments. To validate the device‐level stability of the Cu‐Ag conductor, the optimized ink was integrated as electrodes into solar cells for a comprehensive evaluation of power conversion efficiency (PCE). The resulting devices achieved energy conversion efficiencies comparable to those fabricated with commercial silver electrodes, demonstrating exceptional operational stability without any detectable performance degradation (Figure 4d). The comparable and stable device performance demonstrates that the Cu‐Ag architecture maintains its electrical integrity without significant oxidation or degradation, thereby confirming its suitability for practical energy devices requiring long‐term operational stability.

To demonstrate the practical applicability of the Cu‐Ag conductors, the optimized ink (Cu:Ag = 1:0.5) was printed onto an Al_2_O_3_ substrate and evaluated as a resistive heater (Figure 5a). The printability of the Cu‐Ag ink was further supported by rheological measurements. As shown in Figure S15, the ink exhibits pronounced shear‐thinning behavior. This rheological response is characteristic of screen‐printable conductive inks and is beneficial for achieving stable and well‐defined printed Cu‐Ag patterns. Upon applying different electrical currents, the printed heater exhibits stable and controllable Joule heating behavior. The steady‐state temperature increases monotonically with increasing current, reaching approximately 200°C at an applied current of 1 A, indicating efficient electrical‐to‐thermal energy conversion and good interparticle connectivity within the printed pattern. The heater shows rapid thermal response and stable temperature plateauing, confirming reliable electrical conduction under elevated thermal conditions. Infrared (IR) thermal imaging taken from the backside of the substrate (Figure 5b) further verifies the temperature distribution. The highest temperature is concentrated in the central region of the printed pattern, while the edges exhibit relatively lower temperatures, consistent with heat dissipation toward the surroundings. Finite‐element simulations performed using COMSOL Multiphysics (Figure 5c) reproduce this spatial temperature profile, confirming that the observed heating behavior arises from the designed conductive geometry and uniform current distribution. To further demonstrate the potential for functional integration, a shape memory alloy (SMA) coil was fabricated via laser cutting. Shape memory alloys (SMAs) operate through a reversible, solid‐state phase transformation between two distinct crystal structures: Martensite (the low‐temperature phase) and Austenite (the high‐temperature phase). Unlike typical metals that undergo permanent (plastic) deformation via atomic slip, SMAs accommodate strain through a process called twinning, which allows the material to recover its original geometry upon thermal activation. The SMA coil was fabricated using laser cutting testing equipment, and placed on top of the printed heater as a simple actuator demonstration (Figure 5d). Upon current injection, the heater temperature increases to the activation threshold of the SMA, triggering its shape transformation and generating mechanical motion. This proof‐of‐concept device demonstrates that the Cu‐Ag conductor can serve not only as a high‐temperature‐stable interconnect but also as an effective electrothermal driving platform. These results collectively illustrate the potential of the developed ink for integrated heating, actuation, and thermally driven electronic systems operating under demanding conditions.

**FIGURE 5:**
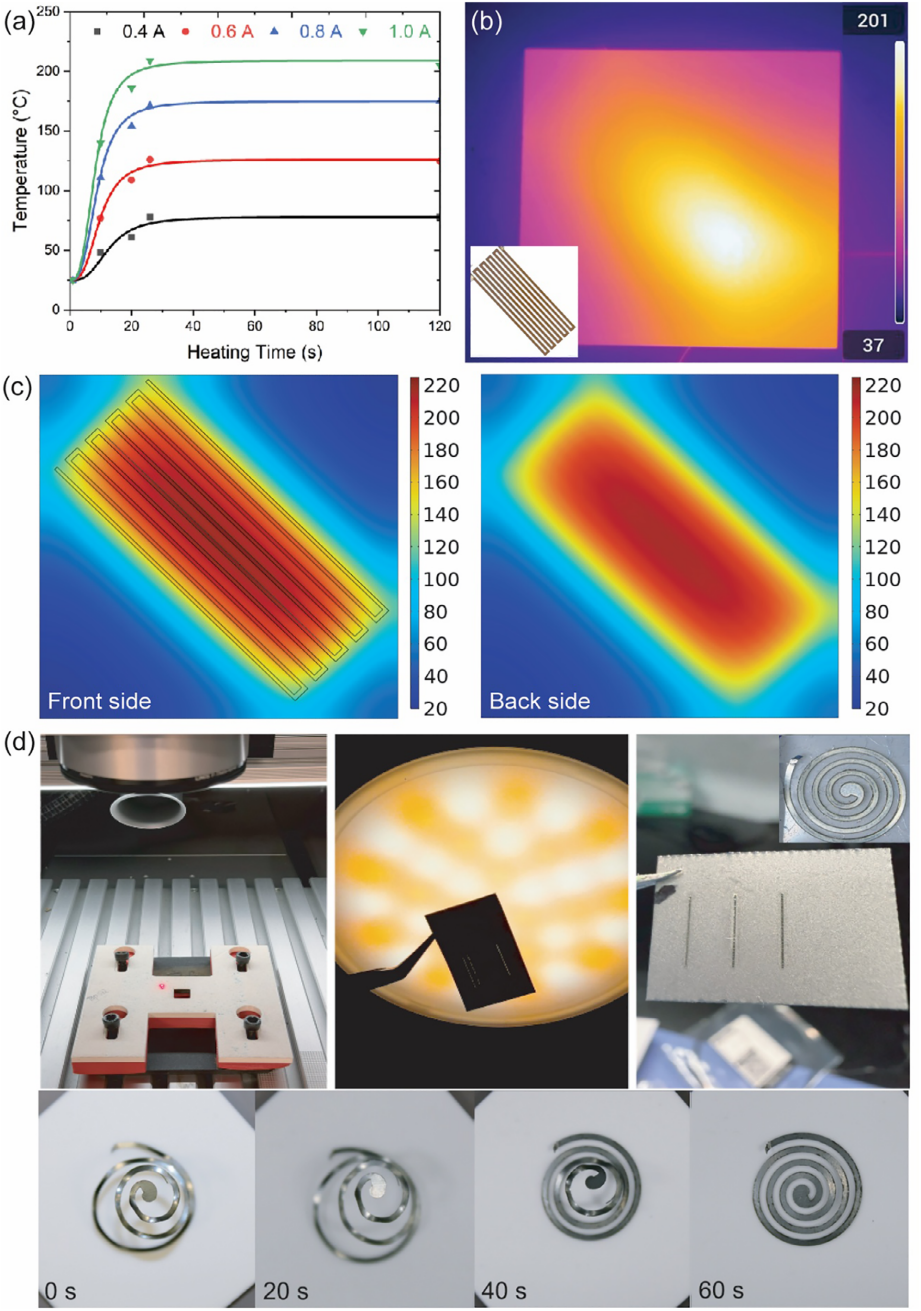
Applications of heater and actuator using Cu‐Ag conductor: (a) The temperature changes with different applied currents for the heater. (b) IR images captured by a thermographic camera functioning as a heater with an applied current of 1 A (the unit is°C). (c) The front and back sides of the calculated thermal distribution of the heater. (d) The testing setup for laser fabrication and the driving process of shape memory metal under the action of a heater.

## 3 Conclusions

In summary, we present a particle‐free, air‐sinterable ink that enables printed copper conductors with exceptional thermal stability through an in‐situ self‐reduction strategy integrating copper and in situ electroless silver deposition, forming a conformal Ag‐surface‐enriched Cu conductive network during low‐temperature sintering at 200°C in air. With an optimized Cu/Ag molar ratio, the printed conductors achieve a conductivity of 4.3 MS m^−^^1^ and maintain stable electrical performance up to 800°C, including 48 h of aging at 700°C in air—surpassing the thermal limits of conventional copper inks and many previously reported core‐shell systems. The engineered Ag shell and robust Cu‐Ag interfaces not only suppress copper oxidation but also mitigate thermally and electrically driven silver migration, addressing key reliability challenges under high‐temperature and high‐humidity operation. This work establishes a scalable and application‐ready pathway toward thermally robust copper conductors for advanced printed electronics and energy systems.

## 4 Experimental Methods

### 4.1 Materials

Copper(II) formate tetrahydrate (Cu(HCOO)_2_, 98%, Thermos Scientific), 2‐Amino‐2‐methyl‐1‐propanol (AMP, 95%, Thermos Scientific), silver nitrate (AgNO_3_, 99%, Sigma‐Aldrich), and DI water are used without further purification. The shape memory alloy (NiTi) was purchased from Amazon.

### 4.2 Fabrication of Inks and Synthesis of Cu‐Ag Conductors

The Cu‐Ag composite inks were prepared by first dispersing copper(II) formate (Cu(HCOO)_2_) in AMP (with a molar ratio of 1:2) to form a homogeneous Cu precursor ink. In parallel, silver nitrate (AgNO_3_) was dissolved in deionized water to obtain an Ag precursor solution. The two precursor components were then combined at different Cu:Ag molar ratios (1:0.1, 1:0.3, 1:0.5, 1:0.7, and 1:0.9) to produce a series of Cu‐Ag mixed inks. The resulting inks were printed onto various substrates, followed by air sintering to convert the printed patterns into conductive Cu‐Ag conductors. Unless otherwise specified, the Cu‐MOD ink and AgNO_3_ aqueous precursor were stored separately in sealed containers at ∼5°C and mixed immediately before screen printing.

### 4.3 Printing of Cu‐Ag Conductive Patterns

The Cu‐Ag conductive patterns were fabricated by screen printing using a stainless‐steel screen with an opening size of approximately 80 µm. During printing, the squeegee angle was maintained at approximately 45–60°, and the printing speed was set to 50–80 mm s^−^^1^. A snap‐off distance of 5 mm was used to ensure uniform ink transfer from the screen to the substrate. After printing, the patterns were dried at 80°C to remove residual solvent, followed by thermal conversion/sintering at the designated temperature.

### 4.4 Characterizations

Thermal stability was assessed by thermogravimetric analysis using an SDT Q600 (TA Instruments, USA) in flowing air with a temperature ramp of 10°C min^−^^1^. Phase identification was carried out on an ARL Equinox 100 diffractometer using a 0.1° step increment. Chemical bonding/functional groups in the Cu–Ag composites were examined by FTIR spectroscopy (Agilent Cary 560). Surface and cross‐sectional microstructures, together with elemental distributions, were characterized by field‐emission SEM (Hitachi SU‐70) coupled with EDS mapping. TEM imaging of aluminum formate was performed on a JEM‐2100 instrument (JEOL Ltd., Japan). Electrical conductivity at room temperature was measured with an Ossila four‐point probe system. For elevated‐temperature electrical stability, resistance/conductivity was recorded using a Keithley 2450 SourceMeter while the samples were heated in a laboratory oven serving as the thermal source. A Thermo Scientific HAAKE MARS iQ Rotational Rheometer was utilized to study the rheological properties of the MOD‐inks to assess their printability.

In the drop‐tower evaluation of the printed conductors, we employed an FR4 cantilever‐beam coupon as the mechanical test vehicle. Copper pads were patterned on the substrate to enable in situ electrical resistance tracking during impact events, and strain gauges were integrated to correlate electrical response with the local deformation of the beam. The printed conductor patterns (air‐sinterable Cu ink and Cu‐Ag composite ink) were intentionally routed toward the fixed end of the cantilever, so that the most critical segments reside in the region of maximum bending strain generated by drop‐induced flexure.

To evaluate electrochemical/ionic migration behavior, printed Ag electrodes and Cu‐Ag electrodes were fabricated in the same test layout and mounted in a humidity and temperature controlled chamber. The samples were exposed to 100% relative humidity while a constant DC electric field of 80 V·cm^−^^1^ was applied between the opposing electrodes. The electrode bias was maintained throughout the test to drive moisture‐assisted metal dissolution/transport and potential dendritic growth across the gap. After testing, the electrode surfaces and the inter‐electrode region were inspected using SEM to assess evidence of dendrite formation and corrosion products, enabling a direct comparison of migration susceptibility between Ag and Cu‐Ag conductors under identical field and humidity conditions.

Commercial aluminum‐back‐contact solar cells (Al‐BSF type) were used as the device platform. Prior to printing, the cell surface in the contact region was cleaned to remove loose contaminants. The as‐prepared Cu‐Ag ink was then printed directly onto the locations of the original front‐side electrodes/gridlines to form the current‐collecting contact. After printing, the patterned Cu‐Ag traces were air‐sintered to generate a conductive Cu‐Ag network and establish electrical connection to the underlying contact layer. External leads were subsequently attached to the printed conductors through an ohmic contact, enabling electrical characterization of the solar cells using the printed Cu‐Ag electrodes.

The heater patterns were fabricated by screen printing, followed by air sintering at 200°C to convert the printed features into conductive traces. After sintering, electrical leads were attached to the contact pads and the heater performance was evaluated by applying a DC bias with a Keithley 2450 SourceMeter, and monitoring the resulting temperature rise using a thermographic infrared camera. A Trotec SpeedMarker 700 fiber laser system (30 W, 1064 nm wavelength) was utilized to evaluate the fabrication of coils from 0.3 mm thick Shape Memory Alloy (SMA) sheets (Nexmetal Corp.) which have a 50°C phase transition temperature. The helical coil was fabricated from the SMA sheet using the fiber laser. The design features a width of 1.5 mm for the conductive traces in a planar spiral a starting radius of 2 mm and pitch of 3.3 mm and a total of four turns. The laser cutting was performed using a multi‐pass approach to ensure clean edge quality on the SMA sheet. Specifically, the process used 200 passes at a scan speed of 520 mm/s and a pulse frequency of 20 kHz to cut through the 0.3 mm thick SMA.

## Author Contributions

**Qikun Feng**: conceptualization, methodology, investigation, validation, formal analysis, visualization, writing – original draft, writing – review and editing, data curation, software. **Dennis Zhao**: methodology, investigation, validation, formal analysis, visualization. **Samuel von Keitz**: methodology, investigation, formal analysis. **Chris A. Martinez**: investigation, validation, formal analysis. **Abhijit Dasgupta**: supervision, funding acquisition, project administration, writing – review and editing. **Hanyu Liu**: methodology, investigation, validation, visualization. **Zhongxuan Wang**: investigation, validation, formal analysis. **Minhazur Rahman**: investigation, validation, formal analysis, visualization. **Nathan Lazarus**: supervision, funding acquisition, writing – review and editing, project administration. **Shenqiang Ren**: conceptualization, formal analysis, methodology, supervision, funding acquisition, writing – original draft, writing – review and editing, project administration, resources.

## Funding

Air Force Research Laboratory (AFRL) with SEMI‐FlexTech under Agreement Number FA8650‐20‐2‐5506 in support of Army Research Laboratory (ARL).

## Conflicts of Interest

The authors declare no conflicts of interest.

## Supporting information




**Supporting File 1**: advs78439‐sup‐0001‐SuppMat.docx.

**Supporting File 2**: advs78439‐sup‐0002‐VideoS1.mp4.

## Data Availability

The data that support the findings of this study are available from the corresponding author upon reasonable request.
