# Supplementary material for: Molecular Conversion of Thermally Stable Cu‐Ag Networks for Mechanically Robust Printed Electronics
Source: Adv Sci (Weinh). 2026 Oct 25:e78439. Online ahead of print. doi: 10.1002/advs.78439 (PMC13696876; doi:10.1002/advs.78439)
Supplement: Supplementary file 1 [file ADVS-9999-e78439-s001.docx]

Supporting Information

Molecular Conversion of Thermally Stable Cu-Ag Networks for Mechanically Robust Printed Electronics

Qikun Feng^1^, Dennis Zhao^1^, Samuel von Keitz^1^, Chris A. Martinez^2^, Abhijit Dasgupta^2^, Hanyu Liu^1^, Zhongxuan Wang^1^, Minhazur Rahman^3^, Nathan Lazarus^3^, Shenqiang Ren^1^*

^1^Department of Materials Science and Engineering, University of Maryland, College Park, Maryland 20742, United States

^2^Department of Mechanical Engineering, University of Maryland, College Park, Maryland, 20742, United States

^3^Department of Electrical and Computer Engineering, University of Delaware, Evans Hall, 139 The Green, Newark, DE, 19716 USA

Email: sren@umd.edu

Figure S1. XRD of air-sintered Cu ink at different temperatures.

Figure S2. Electrical conductivity of air-sintered Cu ink at different sintering temperatures in air.

Figure S3. Cross-sectional morphologies and corresponding energy dispersive X-ray spectroscopy (EDX) images of Cu-Ag conductor with a molar ratio of 1:0.1.

Figure S4. Cross-sectional morphologies and corresponding energy dispersive X-ray spectroscopy (EDX) images of Cu-Ag conductor with a molar ratio of 1:0.5.

Figure S5. Cross-sectional morphologies and corresponding energy dispersive X-ray spectroscopy (EDX) images of Cu-Ag conductor with a molar ratio of 1:0.9.

Figure S6. FTIR of copper formate, air-sintened Cu ink, AgNO_3_ and Cu-Ag.

Figure S7. FTIR of Cu-Ag conductor after sintering at 200 ℃ with a molar ratio between Cu and Ag of 1:0.1 and 1:0.9.

Figure S8. SEM and corresponding EDX maps of Cu-Ag conductor with a molar ratio of 1:0.1 after aging at 500℃.

Figure S9. SEM and corresponding EDX maps of Cu-Ag conductor with a molar ratio of 1:0.9 after aging at 500℃.

Figure S10. SEM and corresponding EDX maps of Cu-Ag conductor with a molar ratio of 1:0.1 after aging at 1000℃.

Figure S11. SEM and corresponding EDX maps of Cu-Ag conductor with a molar ratio of 1:0.9 after aging at 1000℃.

Figure S12. The setup of the drop-tower tester: The tower consists of a drop table mounted onto 2 guide rods, a seismic base, and the Dual Mass Shock Amplifier (DMSA). Programmer and cardboard are used as pulse-shaping materials. DMSA is used to reach accelerations 20x higher than on the drop table/near the DMSA base. The drop tower test was used to evaluate the electrical reliability of the printed conductor under transient mechanical shock/high-acceleration impact conditions, rather than as the sole evidence for mechanical robustness.

Figure S13. Electromechanical stability of the printed conductor under mechanical deformation.

Figure S14. The migration of Ag trace at 80 V/cm and 100% humidity.

Figure S15. The rheological properties of the Cu-Ag inks.

Table S1. Comparison of representative Cu–Ag printed conductors

| No | Type | Conductivity  (MS/m) | High-temperature/oxidation performance | References |
| --- | --- | --- | --- | --- |
| 1 | Cu–Ag hybrid ink | ~29 | Up to 180 °C in air | S1 |
| 2 | Particle-free Cu–Ag complex ink | ~0.36 | Up to 140 °C in air | S2 |
| 3 | Cu core/Ag shell | N/A | 220 °C in air | S3 |
| 4 | Cu@Ag core–shell submicron particles | ~9.3 | 250 °C in air | S4 |
| 5 | Cu@Ag core–shell nanoparticle ink | ~3.92 | Air-stable at room temperature | S5 |
| 6 | Cu/Ag nanoplatelet | ~2.8 | 160 °C for 98 h in air | S6 |
| 7 | Cu–Ag powder | N/A | 300 °C in air | S7 |
| 8 | Cu@Ag particles for printable paste | ~9.45 | 300 °C in air | S8 |
| 9 | Cu-Ag core-shell nanoparticles | ~3.69 | ~350 °C in air | S9 |
| 10 | Cu@Ag alloy nanowires | 35 Ω/sq | 140 °C for 500 h in air | S10 |
| This work | Particle-free Cu–Ag reactive ink | 4.3 | Stable up to 800 °C in air |  |

References:

S1. Li, W.; Hu, D.; Li, L.; Li, C.F.; Jiu, J.; Chen, C.; Ishina, T.; Sugahara, T.; Suganuma, K. Printable and Flexible Copper–Silver Alloy Electrodes with High Conductivity and Ultrahigh Oxidation Resistance. *ACS Appl. Mater. Interfaces* **2017**, *9* (29), 24711–24721.

S2. Li, W.; L, C.F.; Lang, F.; Jiu, J.; Ueshima, M.; Wang, H.; Liu, Z. Q.; Suganuma, K. Self-catalyzed copper–silver complex inks for low-cost fabrication of highly oxidation-resistant and conductive copper–silver hybrid tracks at a low temperature below 100 °C. *Nanoscale* **2018**, *10* (11), 5254–5263.

S3. Li, W.; Lang, F.; Liang, C.; Tong, K.; Yang, Y.; Jiu, J.; Zhang, J.; Wang, H.; Suganuma, K. Supersaturated solid-solution interfaces of Cu core/Ag shell structures with enhanced thermal stability and oxidation resistance. *Corros. Sci.* **2022**, *201*, 110269.

S4. Pajor-Świerzy, A.; Farraj, Y.; Kamyshny, A.; Magdassi, S. Air stable copper-silver core-shell submicron particles: Synthesis and conductive ink formulation. *Colloids Surf. A: Physicochem. Eng. Asp.* **2017**, *521*, 272-280.

S5. Li, G.; Yu, X.; Zhang, R.; Ouyang, Q.; Sun, R.; Cao, L.; Zhu, P. Facile Preparation of Monodisperse Cu@Ag Core–Shell Nanoparticles for Conductive Ink in Printing Electronics. *Micromachines* **2023**, *14*, 1318.

S6. Sheng, A.; Khuje, S.; Yu, J.; Petit, D.; Parker, T.; Zhuang, C.G.; Kester, L.; Ren, S. Ultrahigh Temperature Copper-Ceramic Flexible Hybrid Electronics. *Nano Lett.* **2021**, 21 (21): 9279–9284.

S7. Li, J.; Li, Y.; Wang, Z.; Bian, H.; Hou, Y.; Wang, F.; Xu, G.; Liu, B.; Liu, Y. Ultrahigh Oxidation Resistance and High Electrical Conductivity in Copper-Silver Powder. *Sci. Rep.* **2016**, 6, 39650.

S8. Jiang, M.; Sun, Y.; Zhu, J.; Lin, B.; Zhang, X. Confinement synthesis via pH-responsive polyethyleneimine-glutaraldehyde cross-linked networks for Cu@Ag with enhanced conductivity and oxidation resistance for electronic applications. *Chem. Eng. J.* **2025**, 522, 166931.

S9. Yu, X.; Li, J.; Shi, T.; Cheng, C.; Liao, J.; Fan, J.; Li, T.; Tang, Z. A Green Approach of Synthesizing of Cu–Ag Core-Shell Nanoparticles and Their Sintering Behavior for Printed Electronics. *J. Alloys Compd.* **2017**, *724*, 365–372.

S10. Zhang, B.; Li, W.; Noji, M.; Chen, C.; Yang. Y.; Sugahara, T.; Koga, H.; Suganuma, K. Alloying and Embedding of Cu-Core/Ag-Shell Nanowires for Ultrastable Stretchable and Transparent Electrodes. *ACS Appl. Mater. Interfaces* **2019**, *11* (20), 18540–18547.
